# The calcium-ROS-pH triangle and mitochondrial permeability transition: challenges to mimic cardiac ischemia-reperfusion

**DOI:** 10.3389/fphys.2015.00083

**Published:** 2015-03-18

**Authors:** Sabzali Javadov

**Affiliations:** Department of Physiology, School of Medicine, University of Puerto RicoSan Juan, PR, USA

**Keywords:** cardiac ischemia-reperfusion, mitochondria, permeability transition pore, calcium, ROS, pH

Reperfusion of the heart following sustained ischemia is associated with enhanced reactive oxygen species (ROS) production, Ca^2+^ accumulation, and pH_i_ normalization that are the major inducers of mitochondrial permeability transition (mPT). Despite intensive studies, a cause-and-effect relationship between the ROS-Ca^2+^-pH_i_ triangle and mPT has not yet been established (Halestrap et al., 2004; Bernardi, 2013). Initially, several proteins such as VDAC, ANT, and phosphate carrier (PiC) have been suggested as the essential structural components of the mPTP. However, genetic studies from different groups demonstrated that pore opening can occur in the absence of these proteins indicating that they are not involved in the mPTP structure (*reviewed in* Bernardi, 2013; Halestrap and Richardson, 2015). Emerging studies suggest that the mitochondrial F_0_F_1_-ATP synthase or electron transport chain (ETC) complex V is involved in pore formation and may actually play an important role as a structural component of the mPTP (Giorgio et al., 2013; Alavian et al., 2014; Azarashvili et al., 2014; Carraro et al., 2014). In addition to the unknown molecular identity of the mPT pore (mPTP), a lack of *in vitro* models mimicking cardiac ischemia-reperfusion (IR) makes it difficult to elucidate the precise role of mitochondrial ROS, Ca^2+^, and pH_i_ in response to oxidative stress. Mitochondrial ETC complexes I, II, and III are the main sites of ROS (superoxide anion) production (Figure 1). Dysfunction of the complexes induced by cardiac IR enhances ROS which are not efficiently eliminated by the mitochondrial antioxidant system due to high ROS generation and low ROS scavenging. Activity of ETC complexes may be diminished by a number of factors including cardiolipin oxidation, degradation of supercomplexes, alteration of the ion homeostasis/redox potential, etc.

**Figure 1:**
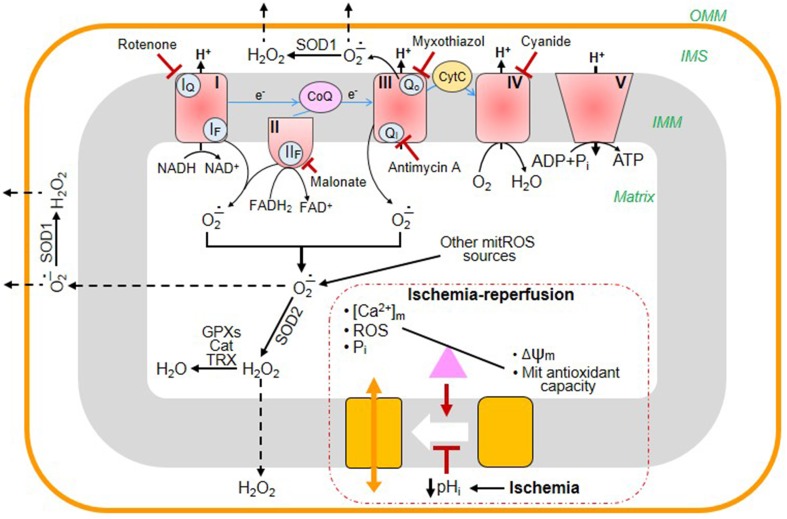
**Main sites of ROS generation in mitochondria and their elimination by the antioxidant system: the effects of cardiac ischemia-reperfusion (IR)**. The mitochondrial electron transport chain (ETC) consists of four multi-subunit complexes (I, II, III, and IV), coenzyme Q (CoQ), and cytochrome c (Cyt C). Electrons (e-) are transferred through the ETC from the reducing NADH/FADH_2_ to O_2_, finally generating H_2_O at complex IV. The electron transfer through the ETC is accompanied by ROS generation at complexes I, II, and III. Complex I oxidizes NADH and produces superoxide (O^−.^_2_) at the flavin site (I_F_) and ubiquinone-binding site (I_Q_) that is released to the matrix. Complex II oxidizes succinate to fumarate and generates O^−.^_2_/H_2_O_2_ both in the reverse and forward reactions through the flavin site (II_F_). Complex III is the main source of ROS that produces O^−.^_2_ to both cytoplasm and matrix. Under normal physiological conditions, the level of mitochondrial ROS is regulated by mitochondrial and cytoplasmic antioxidant systems. Superoxide dismutases (SOD1, SOD2) convert O^−.^_2_ into H_2_O_2_ which is then eliminated by catalase (Cat), glutathione peroxidases (GPXs), and reduced thioredoxin (TRX). During cardiac IR, the balance between generation and scavenging of ROS is altered due to high ROS production and low antioxidant capacity of mitochondria. Mitochondrial permeability transition pores (mPTP) remain closed during ischemia due to low pH_i_. However, at reperfusion, excessive ROS generation together with increased [Ca^2+^] and [P_i_]_m_ and diminished membrane potential (Δψ_m_) cause mPTP opening. The blunted arrows indicate the sites where the inhibitors rotenone, malonate, myxothiazol, antimycin A, and cyanide bind to ETC complexes.

The article published by Lindsay et al. (2015) studies pH-dependence of Ca^2+^-induced swelling (a marker of mPTP opening), ROS generation and respiratory function of isolated guinea pig cardiac mitochondria using substrates and inhibitors for ETC complexes I and III. Results of the study demonstrated that pH and Ca^2+^-induced mPTP opening have different effects on ROS production at complexes I and III. The authors attempted to mimic cardiac IR by blocking complexes I and III with rotenone and antimycin in the presence of pyruvate and succinate, respectively. Although this is the only approach to assess the contribution of individual ETC complexes to ROS production in isolated mitochondria, it is rather different from the *in vivo* condition observed in cardiac IR. Each of complexes I and III contain two sites of ROS generation, and rotenone and antimycin inhibit only one site at complex I (the ubiquinone-binding site, I_Q_) and complex III (the quinone-reducing center, Qi), respectively. Complete chemical blocking of these sites and the use of only one substrate (pyruvate or succinate) for each complex are the major limitations of the study. On the other hand, more recent studies revealed that succinate is a general metabolic marker of ischemia in a variety of tissues including the heart, and that it is responsible for mitochondrial ROS production during reperfusion by reverse electron transport at complex I Inhibition of ischemic succinate accumulation and its oxidation after subsequent reperfusion was sufficient to ameliorate *in vivo* cardiac IR injury in rodents (Chouchani et al., 2014). Indeed, in the study by Lindsay et al. (2015), the authors measured ROS levels at complex I-III, but not complex III alone. Most of the ROS signal observed during succinate oxidation is rotenone-sensitive and this is associated with the I_Q_ site of complex I due to the backflow of electrons from the reduced Q-pool. Accordingly, reverse electron transfer from the reduced QH_2_ pool at site I_Q_ should be blocked to measure ROS generation solely at complex III.

Oxidative stress induces a complex of biochemical, biophysical and topographical changes of the inner mitochondrial membrane that ultimately result in malfunction of the ETC. The latter, when accompanied by membrane depolarization, ROS generation, matrix Ca^2+^ and P_i_ overload, can induce reversible (low conductance, physiological) or irreversible (high conductance, pathological) mPT depending on the severity of IR. Moreover, opening of the mPTP can further enhance the aforementioned alterations. Since low pH_i_ in the ischemic myocardium blocks the mPTP, pore opening occurs only upon reperfusion with normalization of pH_i_ (Griffiths and Halestrap, 1995). The contribution of each ETC complex may be different throughout the ischemic period due to changes in the redox potential, ion homeostasis, and antioxidant system of mitochondria. Mitochondrial respiration, ROS generation and mPTP opening were pH-dependent, which indicates that interactions between these parameters are complex (Lindsay et al., 2015). ROS production at pH 6.5 was significantly lower than that at pH 6.9 and pH 7.15 for complexes I and I-III in mitochondria with Ca^2+^ swelling. Notably, significant mitochondrial swelling associated increased ROS generation was observed in the presence of succinate and antimycin A at all pH (6.5; 6.9, and 7.15), and both swelling and ROS production were significantly reduced by cyclosporin A to basic levels. These data confirm previous studies that mPTP opening induces mitochondrial ROS production (Batandier et al., 2004) and lowering pH inhibits pore opening (Javadov et al., 2008). However some findings of the study remain unanswered. For instance, Ca^2+^-induced swelling was accompanied by a great increase in ROS release from complex I at pH 6.9 but not at pHs 7.15 or 6.5. Respiration rates (state three and RCI) were markedly affected at pHs 6.5 or 6.9 for complex I but not for complex I-III during Ca^2+^ swelling. Remarkably, inhibition of complex I by rotenone blocks PTP opening in tissues that express low levels of cyclophilin D, and, conversely rotenone does not affect the PTP in tissues which are characterized by high levels of expression of cyclophilin D and sensitivity to cyclosporin A (Li et al., 2012). The inhibitory effect of rotenone on PTP can complicate the interpretation of the results reported by Lindsay et al. (2015). In addition, it is very difficult to assess the effect of matrix pH on mPTP *in vitro* to mimic conditions observed *in situ*. First of all, the relevant parameter is matrix pH (Nicolli et al., 1993); in deenergized mitochondria the probability of pore opening has an optimum at matrix pH 7.4, although is changes both below and above this level. During IR, matrix pH is influenced by reenergization, and by the secondary events that follow transport of species that depend on the delta pH. This is critical for the PTP, because reenergization may offset the protective effects of an initially acidic matrix pH because of increased Pi uptake, and as shown in isolated brain mitochondria, ischemic and postischemic acidosis may worsen rather than relieve PTP-dependent tissue damage (Kristian et al., 2001).

In conclusion, despite certain limitations, the elucidation of the contribution of ROS and pH_i_ to mPTP opening via chemical inhibition of complexes I and III by Lindsay et al. (2015) opens new directions for further studies.

## Conflict of interest statement

The author declares that the research was conducted in the absence of any commercial or financial relationships that could be construed as a potential conflict of interest.
